# Wand-Based Calibration Accuracy for Unsynchronized Multicamera Systems Without Timestamps

**DOI:** 10.3390/s26030777

**Published:** 2026-01-23

**Authors:** Yuji Ohshima

**Affiliations:** Faculty of Human Health, Kurume University, 1635 Miichou, Kurume 839-0851, Fukuoka, Japan; ohshima_yuuji@kurume-u.ac.jp

**Keywords:** human motion measurement, motion capture, camera parameter

## Abstract

Motion capture experiments can be conducted more easily if marker-based motion (marker-based MoCap) can be captured using an asynchronous multicamera system (Async MCS). However, camera calibration is essential for marker-based MoCap, and a wand calibration method that utilizes timestamp functions has been proposed for Async MCS. However, in practice, many cameras do not provide accurate timestamp functions, limiting the applicability of existing methods in such environments. A wand calibration method for Async MCS that does not rely on timestamp functions is proposed to evaluate the accuracy of estimated camera parameters. In conventional methods, the time offset in image acquisition is obtained from timestamp information, and synchronous coordinates are estimated by interpolating time-series digitized coordinates of wand markers. In this study, the time offset is treated as an optimization variable, which enables camera parameter estimation without using timestamp functions. Consequently, the three-dimensional reconstruction errors of fixed points obtained using the proposed method are significantly smaller (1.445 ± 0.833 and 1.746 ± 0.908 mm) compared to estimations that ignore time offsets. These findings demonstrate that the proposed method enables more accurate camera calibration.

## 1. Introduction

Motion capture is indispensable in the biomechanical evaluation and analysis of human movement, and various methods such as inertial sensor-based motion capture and optical motion capture using cameras (optical MoCap) have been widely employed. Among the optical MoCap techniques, infrared-camera-based motion capture (IR MoCap) can be used to measure the three-dimensional (3D) coordinates of reflective markers with very high accuracy (errors < 1 mm [1,2]). Consequently, IR MoCap has become the standard measurement technique currently used in biomechanics research [3,4,5,6,7,8]. Matsuo et al. [4] measured the motion of the middle finger during baseball pitching and demonstrated that the proximal and distal interphalangeal joints extended until the instant of ball release. Kotsifaki et al. [3] analyzed the single-leg jump performance of athletes that underwent anterior cruciate ligament reconstruction and were cleared to return to sport. They reported that joint work generated by the involved knee was lower than that generated by the uninvolved knee. Insights related to performance enhancement and injury mechanisms have been reported in previous studies that employed IR MoCap. Although IR MoCap has contributed to the accumulation of biomechanical data, the use of the IR MoCap requires purchasing a system that includes highly expensive infrared cameras and dedicated software, and many research and educational institutions that cannot implement such systems. The author developed a marker-based motion capture system using RGB cameras (RGB MoCap) and demonstrated that three-dimensional coordinates of spherical markers can be reconstructed using an average error of 2.24 mm for addressing this issue [9]. This enabled marker-based motion capture using relatively inexpensive RGB cameras compared with infrared cameras; however, there are still aspects of the proposed RGB MoCap that require improvement. Optical motion capture requires conducting experiments with a time-synchronized multicamera system (Sync MCS) regardless of whether IR MoCap or RGB MoCap is used. Therefore, RGB MoCap cannot utilize less expensive RGB cameras that do not have synchronization capabilities. Synchronizing multiple cameras requires dedicated hardware and cabled connections between each camera, making the overall system extremely large and complex. Consequently, experiments using a Sync MCS are conducted in laboratory settings, and performing motion capture outdoors requires substantial time and effort for the setup. In recent years, many inexpensive, compact RGB cameras that perform high-speed recording have become commercially available. If marker-based motion capture could be performed using videos acquired through asynchronous recording (Async RGB MoCap), its cost-effectiveness and practicality would be greatly improved, thereby addressing the aforementioned limitations.

**Figure 1:**
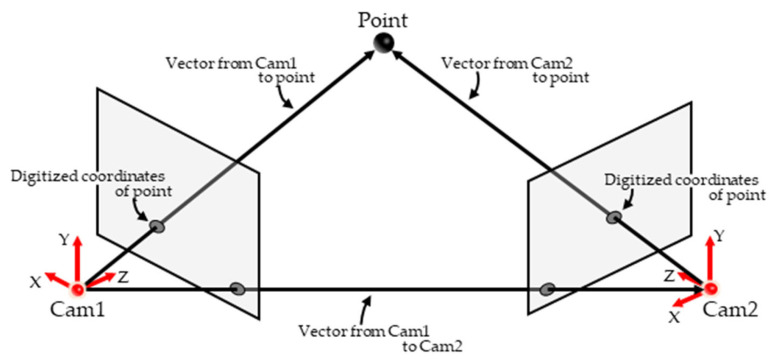
Epipolar geometry.

**Figure 2:**
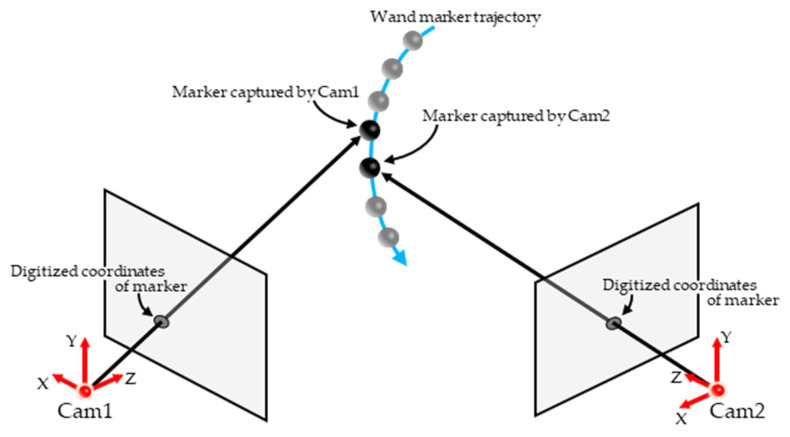
Wand marker captured by an asynchronous multicamera system.

The following process is required to reconstruct the 3D coordinates of measurement points using Optical MoCap. Multiple cameras with known camera parameters (e.g., position, orientation, and focal length) are prepared. The measurement points are recorded by each camera, and the coordinates of the points on the image plane (digitized coordinates) are obtained from captured images. Subsequently, camera rays (half-lines extending from the camera positions toward the measurement points) are determined using camera parameters and digitized coordinates, and the intersections of camera rays from multiple cameras correspond to the three-dimensional coordinates of measurement points. Three-dimensional coordinates are reconstructed through this process, and therefore, the accuracy of camera parameters had a significant effect on the measurement accuracy of optical MoCap. Various methods are proposed to estimate camera parameters, which can be broadly classified into two types. The first type exemplified by direct linear transformation (DLT [10]) estimates camera parameters by placing multiple calibration points with known 3D coordinates within the measurement volume (known points) and using correspondences between digitized coordinates and known 3D coordinates. The second type exemplified by nonlinear transformation (NLT [11]) utilizes calibration points observed by multiple cameras whose 3D coordinates are unknown (unknown correspondence points) and estimates camera parameters based on relationships between the unknown points and camera positions (Figure 1). Both methods estimate camera parameters with high accuracy, and calibration methods that apply these two approaches have been proposed [12,13,14,15,16]. DLT requires placing multiple known points within the measurement volume, which can make the calibration process time-consuming and labor-intensive for the characteristics of the calibration process. In contrast, NLT requires objects with a known size or length must be captured; however, calibration points can be placed arbitrarily so the time and effort required for calibration are often less than those for DLT. In practice, when using IR MoCap, camera parameters are estimated using a simple calibration procedure called wand calibration, wherein a calibration device (wand) with a known distance between two reflective markers is waved randomly within the measurement volume. However, wand calibration is designed for recording with a Sync MCS [17,18]. In an asynchronous multicamera system (Async MCS), unknown correspondence points cannot be captured (Figure 2), which reduces the accuracy of wand calibration.

**Figure 3:**
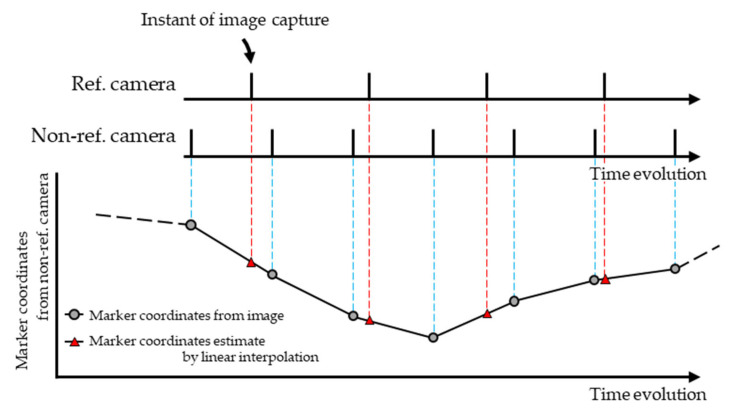
Interpolation method proposed by Zhang and Fu [19].

To address this issue, Zhang and Fu [19] proposed a wand calibration method for Async MCS. In this method, the exact time each frame was captured using the timestamp functions of the cameras, and the temporal offset of the No-ref. cameras relative to the reference camera (Ref. camera) were calculated. The time-series data of digitized co-ordinates of wand markers acquired by the No-ref. cameras were linearly interpolated to estimate digitized coordinates when the Ref. camera captured each image (Figure 3). Subsequently, wand calibration was performed using these estimated digitized coordinates. Although this method improved the accuracy of camera parameter estimation, many cameras cannot record the capture time precisely. For such cameras, the method of Zhang and Fu [19] cannot be applied. In their study [19], the digitized coordinates of the wand markers were obtained using infrared light-emitting diodes and infrared pass filters. However, it remains unclear if digitized coordinates obtained from RGB images via object detection can be used in the same way. Based on this study, it is necessary to propose a method for performing wand calibration from RGB images captured by an Async MCS without relying on the timestamp function to develop an Async RGB MoCap system and evaluate its accuracy.

This study aims to propose a wand calibration method for an Async MCS without relying on the timestamp function and evaluate the accuracy of the estimated camera parameters.

The main contributions of this study include a wand calibration method for an Async MCS that does not rely on the timestamp function, evaluation of the accuracy of camera parameters estimated using the proposed method, and discussion of whether estimated camera parameters provide sufficient accuracy for performing RGB MoCap.

The remainder of this paper is organized as follows: Section 2 describes the experimental procedures, camera parameter estimation process, and accuracy evaluation. Section 3 presents the accuracy results. Section 4 discusses the accuracy of the proposed method and compares it with the accuracy obtained without interpolation. Finally, Section 5 provides the conclusions of this study.

The Async RGB MoCap system being developed by the author is not intended to replace IR-based motion capture systems. Rather, it aims to provide an easy-to-use marker-based motion capture environment for situations in which the introduction of expensive IR-based systems or industrial cameras with hardware synchronization is not feasible due to financial constraints, as well as for outdoor experimental settings where the installation and operation of IR-based systems are difficult.

## 2. Methods

### 2.1. Data Collection

**Figure 4:**
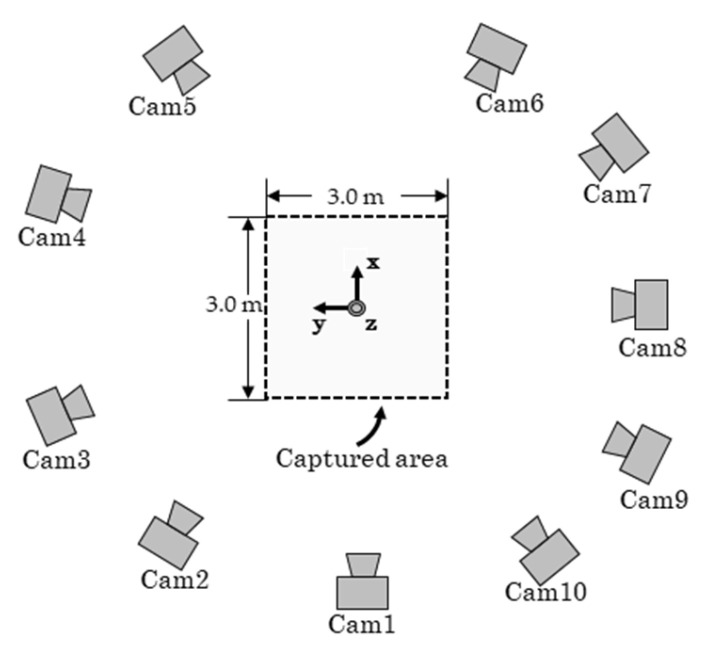
Experimental setup. Ten RGB cameras were arranged to cover a measurement volume of 2.0 m (x) × 4.0 m (y) × 2.0 m (z).

**Figure 5:**
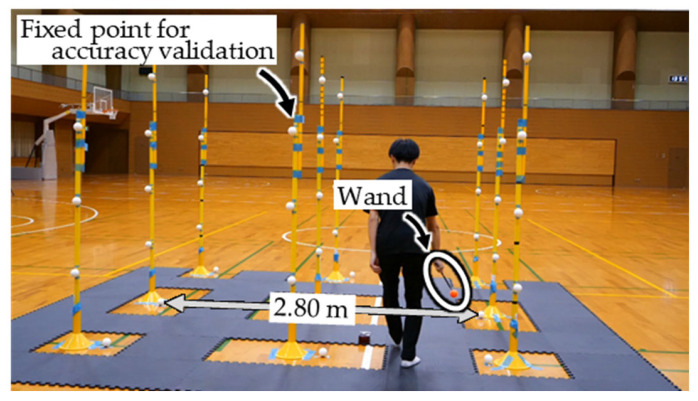
Photograph captured during the experiment. The white spheres represent the fixed points used for accuracy validation.

**Figure 6:**
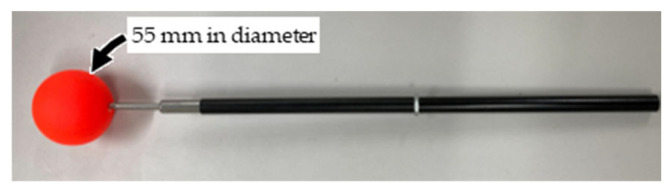
Wand used in the experiment.

**Figure 7:**
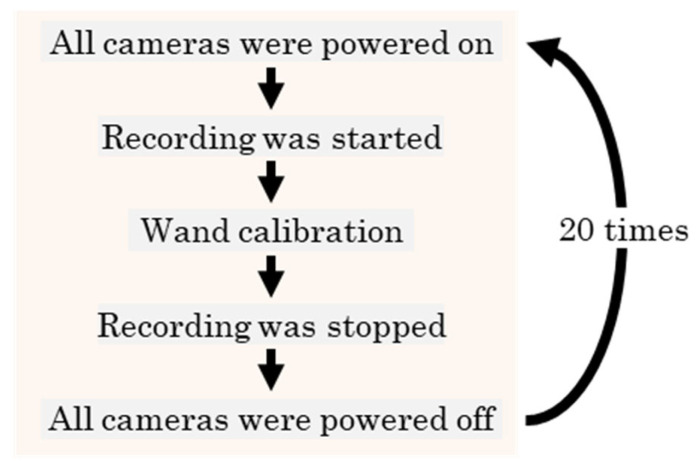
Flow of the 20 recording trials conducted in the experiment.

Figure 4 shows that the measurement volume is set to 3.0 m × 3.0 m × 2.0 m. Ten cameras (LUMIX DC-G9PRO × 6, DC-S1R × 2, DC-BGH1 × 1, DC-GH6 × 1; Panasonic Corporation, Osaka, Japan; frame rate: 180 fps; shutter speed: 1/500 s; resolution: 1920 × 1080 pixels) are used. The positions and focal lengths of the cameras are adjusted such that each camera can capture the entire measurement volume. Within the measurement volume, 45 fixed points are installed for accuracy validation, and two points are placed on the ground level (Figure 5) served as a known length marker (KLM) with an inter-point distance of 2.80 m. An experimenter randomly waved the wand (Figure 6) within the measurement volume, and the motion was recorded asynchronously by the ten cameras. In addition, the image-capture experiment is repeated 20 times using the procedure shown in Figure 7 because the timing offsets between the Ref. Camera and each No-ref. Cameras (Figure 3) are determined randomly.

### 2.2. Gold Standard 3D Coordinates of Fixed Points for Accuracy Validation

Bundle adjustment was performed to determine the gold standard 3D coordinates of 45 fixed points. Each digitized coordinate of 45 fixed points was obtained using a custom digitizer implemented in a numerical computing environment (MATLAB 2021b; Math-Works Inc., Natick, MA, USA). Four arbitrary points on the circumference of each circular marker were digitized. The center of the circle was computed from these coordinates and used as the digitized coordinate of each fixed point. Since the digitization of the fixed points was performed manually by the operator, the correspondence of fixed points across cameras—namely, which fixed point in one camera corresponds to which fixed point in another camera—is known. In the bundle adjustment, camera parameters were estimated through numerical optimization; however, the optimization could converge to a solution far from the true values if improper initial values were used. Therefore, in this study, camera parameters were explored in three stages, as explained below. A similar procedure was used to estimate the camera parameters for the wand calibration.

#### 2.2.1. Stage 1

Extrinsic parameters (camera position and orientation) and focal length were estimated in Stage 1. Lens distortion was not considered at this stage, and the other intrinsic parameters were treated as constants: aspect ratio = 1.0, digitized coordinates of the principal point (intersection of the optical axis and image sensor) = image center (U_0_ = 960.5, V_0_ = 540.5), and skew angle = 0.0°.

**Figure 8:**
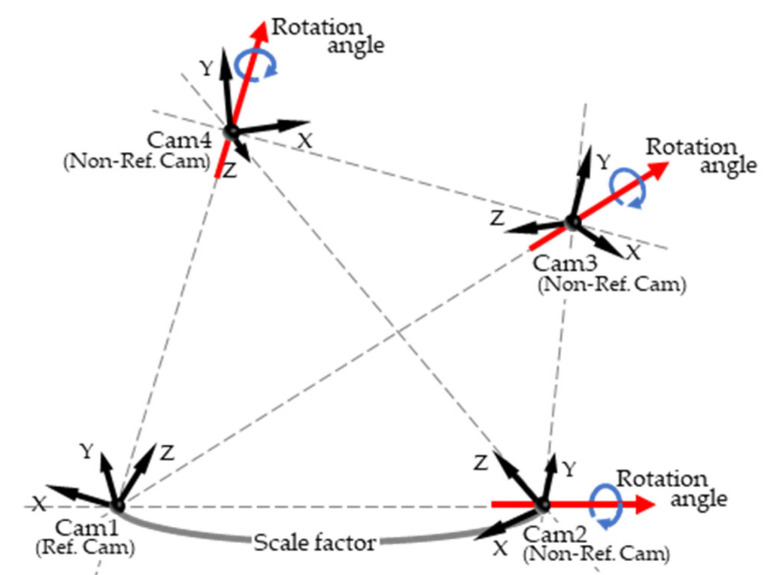
Relative positions and orientations of cameras in the multicamera system. Rotation angle and scale factor are the variables explored in Stage1.

The fundamental matrix F was computed for all pairs of ten cameras using the eight-point algorithm using digitized coordinates of 45 fixed points [20]. F represents a 3 × 3 matrix that indicates the projective geometric relationship between two cameras. When the intrinsic parameters are known, the essential matrix E can be derived from F, and the relative orientation of one camera with respect to the other can be uniquely determined from E [21]. In contrast, with respect to camera position, only the line connecting the two cameras can be determined, whereas the distance between the cameras cannot be determined [21]. Therefore, for each Non-Ref. Camera, if E with respect to the Ref. Camera is known, the relative orientation with respect to the Ref. Camera can be uniquely determined (Figure 8). With regard to camera position, if E between the Ref. Camera and each Non-Ref. Camera, as well as those between Non-Ref. Cameras (e.g., Cam2 and Cam3 in Figure 8) are known; the positions of all cameras can be determined once the distance between an arbitrary pair of cameras among the ten cameras (i.e., the scale factor in Figure 8) is given.

**Figure 9:**
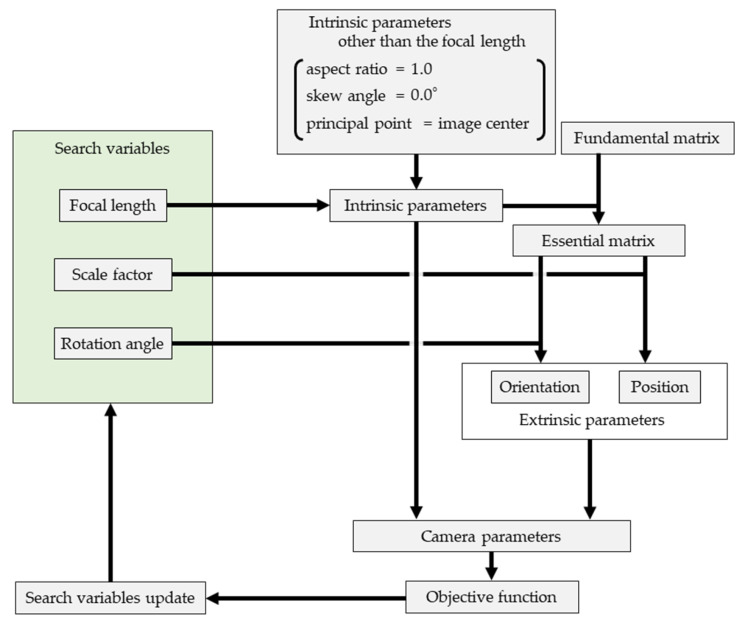
Flowchart of Stage 1 numerical optimization.

**Figure 10:**
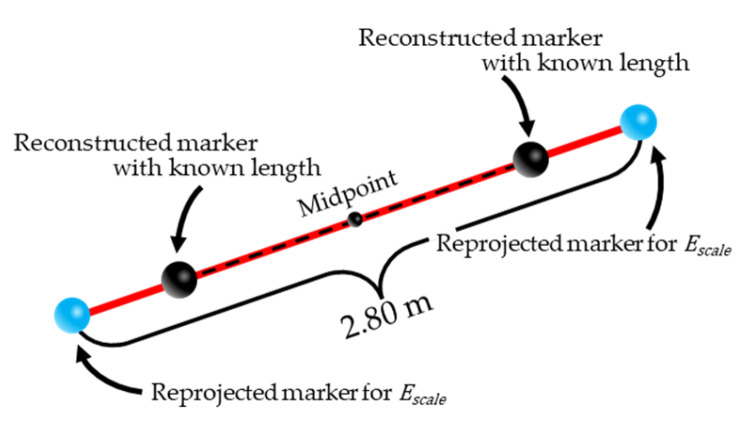
Schematic of the relationship between the reconstructed KLM and line segments used to compute $E_{scale}$.

In Stage 1, all intrinsic parameters except the focal length were treated as constants, and F was known. Thus, in theory, the extrinsic parameters of the cameras could be uniquely recovered if the scale factor and focal length are known. Therefore, the focal lengths and extrinsic parameters of all cameras can be determined with fewer variables by searching for the focal lengths of each camera (ten variables) and scale factor (one variable) that minimizes the objective function J in Equation (1). However, the precision of the digitized coordinates of the 45 fixed points affected results when numerical optimization was actually performed; this caused the objective function value to be higher than expected. To address this, the rotation angles of the Non-ref. cameras around the axes pointing from the Ref. camera to each Non-ref. camera (9 variables) were included as search variables in the numerical optimization (Figure 9).

(1) $$J=E_{reproj}+E_{scale},$$

$$E_{reproj}=\sum_{j=1}^{10}\sum_{i=1}^{45}\left\|x_{ij}^{reproj}-{\hat{x}}_{ij}^{reproj}\right\|,$$

$$E_{scale}=\sum_{j=1}^{10}\sum_{i=1}^{2}\left\|x_{ij}^{scale}-{\hat{x}}_{ij}^{scale}\right\|,$$

where $E_{reproj}$, $x_{ij}^{reproj}$, and ${\hat{x}}_{ij}^{reproj}$ represent the reprojection error of the fixed points, manually digitized coordinates of fixed point i in camera j, and digitized coordinates of fixed point i reprojected onto camera j from the 3D coordinates reconstructed using the camera parameters currently being optimized, respectively. Further, $E_{scale}$, $x_{ij}^{scale}$, and ${\hat{x}}_{ij}^{scale}$ represent the error related to the scale factor, manually digitized coordinates of the KLM for camera j, and digitized coordinates reprojected onto camera j for the two endpoints of the 2.8 m line segment, respectively, where the midpoint and orientation of the line segment are set to match the line connecting the two KLMs reconstructed using the camera parameters being optimized (Figure 10).

The numerical optimization was performed using the Newton method. The initial values were set as follows: the scale factor, rotation angles, and focal lengths were 10.0 m, 0.0°, and randomly chosen within the range of 2500–4200 pixels, respectively. However, the optimization was performed multiple times following the procedure described below because a single optimization run did not sufficiently reduce the objective function:

1. Two points were selected randomly from the fixed points excluding the KLM and removed from the calculation of $E_{reproj}$.
2. The optimization was performed using remaining fixed points to obtain a solution.
3. The obtained solution was used as the initial value for the next optimization.
4. Steps 1–3 were repeated until the mean distance between the reconstructed 3D coordinates of the fixed points and camera rays of each camera was less than 10 mm.

By exploring the variables in this manner, the external parameters of each camera can be estimated stably and with high accuracy, without the need to set detailed initial values.

#### 2.2.2. Stage 2

In Stage 2, the camera positions, orientations, and focal lengths are optimized again; however, in this stage, the camera positions and orientations are each treated as having three degrees of freedom for numerical optimization. The number of variables to be optimized is seven per camera, which totals 70 for all ten cameras, with the initial values taken from the solutions obtained in Stage 1. The objective function and optimization procedure are the same as in Stage 1, and numerical optimization using the Newton method was repeated ten times. In the 10th optimization, $E_{reproj}$ was calculated using all fixed points.

#### 2.2.3. Stage 3

In Stage 3, the focal lengths and extrinsic camera parameters obtained in Stage 2 were treated as constants, whereas the aspect ratio, principal point coordinates, skew angle, and lens distortion coefficients were estimated using numerical optimization. The objective function and optimization procedure were the same as those in Stage 2, and both radial and tangential lens distortions were considered [22]. The 45 fixed points were reconstructed using camera parameters obtained at the end of Stage 3, and their 3D coordinates were designated as the gold standard.

### 2.3. Wand Calibration with an Asynchronous Multicamera System

**Figure 11:**
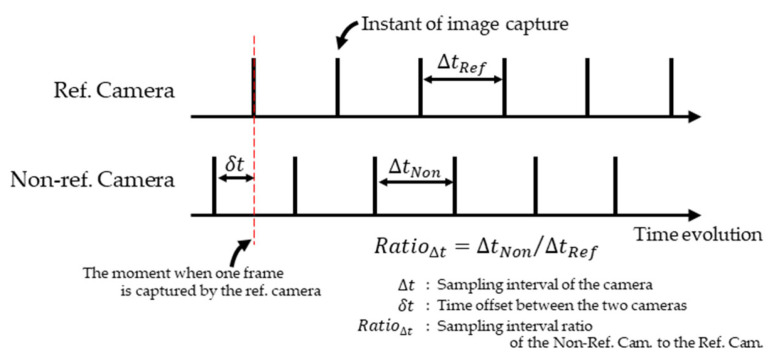
Illustration of variables explored in Stages 2 and 3 (δt and ${Ratio}_{\Delta t}$).

In wand calibration using a Sync MCS, wand markers can be captured simultaneously by all cameras, which enables camera parameters to be estimated by computing $E_{reproj}$ in Equation (1) from the digitized coordinates of the wand markers. However, in this study, an Async MCS is used so that the 3D coordinates of the wand markers differ even in images with the closest recording times across cameras (Figure 2). This can result in the reduced accuracy of wand calibration. The temporal offset in the image capture in an Async MCS arises from differences in the recording start times (δt in Figure 11) and differences in frame rates (Δt in Figure 11). Therefore, if δt, Δt, and the digitized coordinates at non-integer frames (sub-frame) are known, the digitized coordinates of the unknown correspondence points can be obtained, and wand calibration can be performed with the same accuracy as in a Sync MCS.

**Figure 12:**
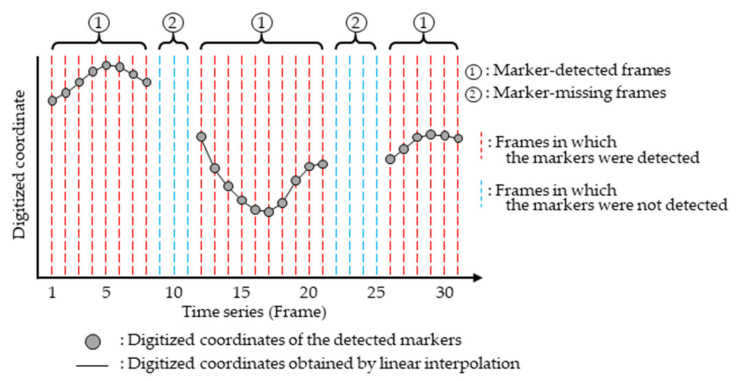
Schematic of the linear interpolation method applied to the time-series data in this study.

In this study, following Zhang and Fu [19], linear interpolation was applied to the discrete time-series data of the digitized coordinates of wand markers obtained from images to estimate digitized coordinates as a continuous function of time (Figure 12). During numerical optimization, the temporal offset of the recording start time of the Non-ref. Camera relative to the Ref. Camera (δt in Figure 11) and frame rate (${Ratio}_{\Delta t}$ in Figure 11) were included as variables, enabling camera parameters to be estimated while accounting for the temporal offset of image capture. In the wand calibration method proposed by Zhang and Fu [19], the image capture time was obtained using the timestamp functions of the camera. In contrast, in the method described above, the image capture time was estimated through numerical optimization, which enabled camera parameters to be determined without using the timestamp function. In this study, the frame rate of each camera was set to 180 fps. However, slight differences in frame rates were observed depending on the camera model (for example, over a 30 min recording, ~40 frames difference occurred between the Lumix GH6 and Lumix BGH1), and therefore, ${Ratio}_{\Delta t}$ was included as a variable in the optimization. In addition, with respect to the interpolation method, we have confirmed that nearly identical results were obtained even when a cubic natural spline function was used.

Wand calibration requires obtaining the digitized coordinates of wand markers. Therefore, a marker-detection neural network was built based on Ohshima’s method [9]. The training epochs, batch size, and pretrained model were identical to those used in Ohshima [9]. For wand calibration, wand markers were placed throughout the entire measurement volume, which takes ~1.5–2.0 min and captures roughly 15,000–20,000 frames.

#### Numerical Optimization

In wand calibration, the camera parameters are estimated through numerical optimization following the same bundle adjustment procedure used to obtain the gold standard described above. The difference is that the reprojection error $E_{reproj}$ in Equation (1) and fundamental matrix F were computed using the digitized coordinates of wand markers. In addition, multiple digitized coordinates of the wand markers are required to compute F**.** Therefore, the moment when the LED light in the measurement volume turned on was visually identified in the video of each camera, and the frame at that instant was used as the image captured at the closest time. The fundamental matrix F was computed using this provisional time synchronization. Both ${Ratio}_{\Delta t}$ and δt were optimized in Stages 2 and 3, whereas in Stage 1, numerical optimization was performed under the provisional temporal synchronization. The initial value of ${Ratio}_{\Delta t}$ was set to 1.00, and δt was defined as the temporal offset relative to the provisional time synchronization and initialized to 0.00 s. In the bundle adjustment for the gold standard, numerical optimization was performed multiple times in each stage. Accordingly, in the wand calibration, we repeatedly performed numerical optimization using only digitized coordinates whose frame numbers had the same last digit as a randomly selected integer from 0 to 9. This means that each numerical optimization uses digitized coordinates obtained from approximately 1500–2000 frames. Then, the 3D coordinates of the fixed points were reconstructed using camera parameters obtained through numerical optimization.

### 2.4. Accuracy Validation

The distance from the 3D coordinates of the 45 fixed points reconstructed by wand calibration to the gold standard was defined as the 3D reconstruction error, and the distance from reconstructed points to the camera rays was defined as the ray distance error. Wand calibration was performed without interpolation to evaluate the effect of interpolation, where ${Ratio}_{\Delta t}$ was set to 1.00 and δt was rounded to the nearest integer of the optimized value for each Non-ref. Camera, and the two errors were calculated. Here, wand calibration with interpolation is referred to as Interp, and wand calibration without interpolation is referred to as No-interp. The capture experiment was conducted 20 times, and a paired t-test was performed to examine differences between the Interp and No-interp data. Furthermore, the same data were calculated using five (Cam1, 3, 5, 6, and 8 in Figure 4) and three cameras (Cam1, 5, 6 in Figure 4), and the paired t-tests were performed.

A 3D reconstruction error was used to evaluate the accuracy of the reconstructed 3D coordinates of markers and serves as a direct metric to assess the precision of marker-based MoCap. The ray distance error is an important metric in marker-based MoCap. The correspondence of markers detected in each camera is initially unknown when reconstructing 3D coordinates from digitized coordinates of spherical markers obtained from multiple cameras. Combinations for which the distance from reconstructed points to each camera ray is below a certain threshold (e.g., 5 mm in Ohshima [9]) must be identified to determine marker correspondences. If the ray distance error is high, accurately determining correspondences becomes difficult, and this makes it a critical metric to evaluate the performance of marker-based MoCap.

## 3. Results

**Figure 13:**
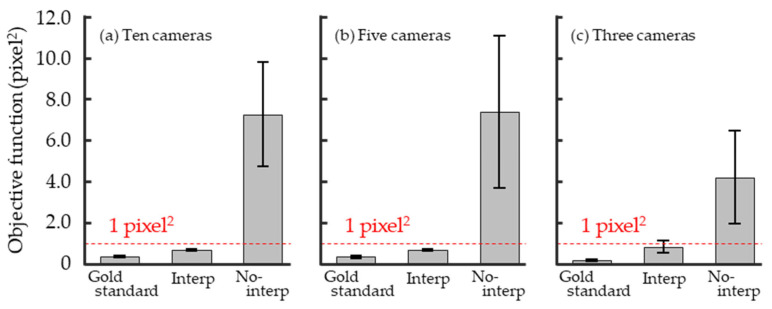
Bar plot of the objective function with standard deviations. Data obtained using multicamera systems with (**a**) ten, (**b**) five, and (**c**) three cameras.

Figure 13 shows the mean and standard deviation of the objective function per camera across 20 trials. The mean values for the gold standard and Interp were below 1 pixel^2^ regardless of the number of cameras used (10, 5, or 3 cameras). For No-interp, the mean values were larger compared to the other two, and some trials exceeded 10 pixel^2^.

**Figure 14:**
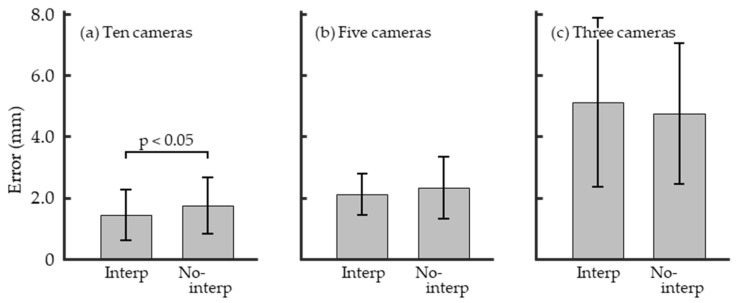
Bar plot of the 3D reconstruction error with standard deviations. Data obtained using multicamera systems with (**a**) ten, (**b**) five, and (**c**) three cameras.

Figure 14 and Table 1 show the results of the 3D reconstruction error. When using ten cameras, the mean value for Interp was 1.445 mm, whereas that for No-interp was 1.747 mm, which shows a significant difference. With five cameras, the mean values were 2.113 mm for Interp and 2.324 mm for No-interp, with no significant difference observed. Similarly, with three cameras, the mean values were 5.121 mm for Interp and 4.745 mm for No-interp; no significant difference was found.

**Figure 15:**
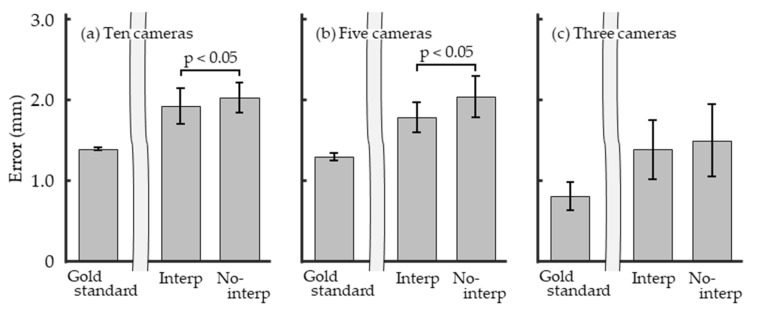
Bar plot of the ray distance error with standard deviations. Data obtained using multicamera systems with (**a**) ten, (**b**) five, and (**c**) three cameras.

Figure 15 and Table 2 present results of the ray distance error. When using ten cameras, the mean value for Interp was 1.914 mm, and for No-interp, it was 2.024 mm, indicating a significant difference. When using five cameras, the mean values were 1.777 mm for Interp and 2.030 mm for No-interp, and a significant difference was observed. In the case of three cameras, the mean values were 1.381 mm for Interp and 1.493 mm for No-interp, and no significant difference was observed.

## 4. Discussion

This study aims to propose a wand calibration method for an asynchronous multcamera system without using the timestamp function and evaluate the accuracy of estimated camera parameters. In this study, the gold standard of the 3D coordinates of the 45 fixed points was determined through bundle adjustment. The 3D coordinates of the 45 fixed points were reconstructed using camera parameters obtained via numerical optimization, which resulted in a ray distance error of 1.389 mm (Table 1). Measurement accuracy can decrease because of soft tissue wobbling when performing motion analysis with markers attached to the body surface. Cereatti et al. [23] investigated such errors and reported that mean errors were ~5–15 mm. Further, there is a high likelihood of human error when the marker attachment is performed manually. Considering these factors alongside the Ray distance error obtained in this study, it is highly probable that the gold standard reconstructed in this study provides reliable 3D coordinates.

For the average value of the objective function per camera, the mean value of the Interp is low regardless of the number of cameras used (Figure 13). In contrast, the mean value for No-interp is higher, and some trials exceed 10.0 pixel^2^ (Figure 13). This indicates that linearly interpolating the digitized coordinates of wand markers and including the frame rate (${Ratio}_{\Delta t}$) and temporal offset at the start of recording (δt) relative to the Ref. Camera as optimization variables can help correct the temporal offset of the image capture.

For the mean 3D reconstruction error using ten cameras, Interp and No-interp are is 1.445 and 1.757 mm, respectively (Table 1). Based on the error data reported by Cereatti et al. [23], it can be considered that in both Interp and No-interp conditions, the 3D coordinates were reconstructed with sufficient accuracy for motion analysis. The mean ray distance errors are 1.914 and 2.024 mm for Interp and No-interp, respectively (Table 2). Ohshima [9] developed RGB MoCap and set a threshold of 5 mm for associating detected spherical markers across cameras. In this study, all obtained ray distance errors were below this threshold, indicating sufficient accuracy for motion analysis. Therefore, it can be concluded that sufficiently accurate camera parameters were estimated under both Interp and No-interp conditions. However, the error is significantly smaller for Interp when comparing the two conditions (Table 1). The information obtained from the experiment (digitized coordinates of wand markers and KLM) used for estimating the camera parameters was common to both conditions. Thus, linear interpolation should be applied to account for the temporal offset of image capture when performing wand calibration with an Async MCS.

There are two reasons why camera parameters can still be estimated with sufficient accuracy in the No-interp condition. In the wand calibration conducted in this study, the wand markers were moved throughout the entire measurement volume in the vertical, anterior–posterior, and lateral directions. Consequently, the positional deviations of unknown correspondence points caused by the temporal offset of image capture were not biased in any specific direction; this can be considered the first reason. Further, Jatesiktat et al. [24] indicated that insufficient accuracy of digitized coordinates or uneven distribution of the captured wand markers within images can lead to a significant decrease in the accuracy of camera parameters when both the extrinsic and intrinsic camera parameters are optimized simultaneously. In the numerical optimization performed in this study, the extrinsic parameters and focal length of each camera were optimized, and then, the remaining camera parameters were optimized. This procedure reduced the degrees of freedom in the numerical optimization; the calibration accuracy was not substantially compromised even if errors caused by temporal offsets in image capture existed.

When comparing the number of cameras used for recording with the mean 3D reconstruction error, the errors were 1.445 mm with 10 cameras, 2.113 mm with 5 cameras, and 5.121 mm with 3 cameras (Interp in Table 1). These results suggest that using a larger number of cameras leads to smaller reconstruction errors. Furthermore, focusing on the increase in error associated with the reduction in the number of cameras, the mean error increased by 0.672 mm when the number of cameras was reduced from 10 to 5, whereas it increased by 3.008 mm when reduced from 5 to 3 cameras. Based on these findings, when estimating camera parameters using the Wand Calibration employed in this study, at least five cameras are likely required. In the present Wand Calibration, the camera parameters were estimated using only the minimum necessary information (digitized coordinates of wand markers and the two KLM points). Therefore, when fewer than five cameras are available, it would be necessary to supplement the calibration information by using a wand with a known distance between two markers (1D wand) or a wand with known geometric structure (2D or 3D wand). In the comparison between the Inter and No-interp, a statistically significant difference was observed only in the ten-camera configuration (Table 1), suggesting that the proposed method may be more effective under conditions with sufficient camera redundancy.

## 5. Conclusions

In this study, we proposed a wand-calibration method compatible with an Async MCS without relying on timestamp functions and evaluated the accuracy of the estimated camera parameters. The results confirmed that interpolating the digitized coordinates of wand markers and compensating for the temporal misalignment of image recording significantly reduced the errors. However, the camera parameters were estimated with sufficient accuracy even without accounting for temporal offsets, which suggests that the spatial distribution of wand markers throughout the measurement volume and numerical optimization procedures substantially affect the accuracy of parameter estimation.

## 6. Patents

The author intends to file a patent application related to the method described in this manuscript.

## Figures and Tables

**Table 1:** Statistics of the 3D reconstruction errors.

Number of Cameras	Interp (Mean ± SD)	No-Interp (Mean ± SD)
Ten cameras	1.445 ± 0.833 mm *	1.747 ± 0.908 mm
Five cameras	2.113 ± 0.662 mm	2.324 ± 1.019 mm
Three cameras	5.121 ± 2.753 mm	4.745 ± 2.304 mm

*: *p* < 0.05 compared to No-interp.

**Table 2:** Statistics of the ray distance errors.

Number of Cameras	Gold Std. (Mean ± SD)	Interp (Mean ± SD)	No-Interp (Mean ± SD)
Ten cameras	1.389 ± 0.012 mm	1.914 ± 0.222 mm *	2.024 ± 0.186 mm
Five cameras	1.290 ± 0.047 mm	1.777 ± 0.185 mm *	2.030 ± 0.256 mm
Three cameras	0.810 ± 0.173 mm	1.381 ± 0.367 mm	1.493 ± 0.448 mm

*: *p* < 0.05 compared to No-interp.

## Data Availability

The data presented in this study are available upon request from the corresponding author.

## Abbreviations

The following abbreviations are used in this manuscript:

Async MCS	Asynchronous multicamera system
DLT	Direct linear transformation
IR MoCap	Infrared-camera-based motion capture
KLM	Known length marker
NLT	Nonlinear transformation
Optical MoCap	Optical motion capture using cameras
RGB MoCap	Marker-based motion capture system using RGB cameras
Sync MoCap	Time-synchronized multicamera system
